# AfroSHARE Summit: visions of health equity for black women with gynecologic and breast cancers

**DOI:** 10.1016/j.gore.2026.102208

**Published:** 2026-09-15

**Authors:** Nefa-Tari Moore, Megan-Claire Chase, Christine Benjamin, Stephanie Poland, Kaiyun Chen, Denise Lawson, Jennel Zeppieri, Leslie R. Boyd, Jane L. Snowdon

**Affiliations:** a SHARE Cancer Support, 315 W 39th Street, Suite 910, New York, NY 10018, USA; b New Women New Yorkers, 547 LaGuardia Place, New York, NY 10012, USA; c People Overcoming and Persevering (Pop) Services, 261 Hillside Ave., White Plains, NY 10603, USA; d Department of Medical Oncology, Weill Cornell Medicine, 530 East 70^th^ Street, M-522, New York, NY 10021, USA; e New York University Grossman School of Medicine, NYU Langone Health, Laura and Isaac Perlmutter Cancer Center, Department of Obstetrics and Gynecology, Division of Gynecologic Oncology, 240 E 38th St 20th Floor, New York, NY 10016, USA; f Independent Researcher, 78 River Road, Cos Cob, CT 06807, USA

## Abstract

• Black voices across the gynecologic/breast cancer care continuum recommended ways to deliver services more effectively.
• Gynecologic/breast cancer survival inequities narrow when patients get guideline-concordant therapy and equal trial access.
• Self-care and self-advocacy are essential for cancer journey navigation from screening to physical and emotional needs.

## Graphical abstract

**Figure:**
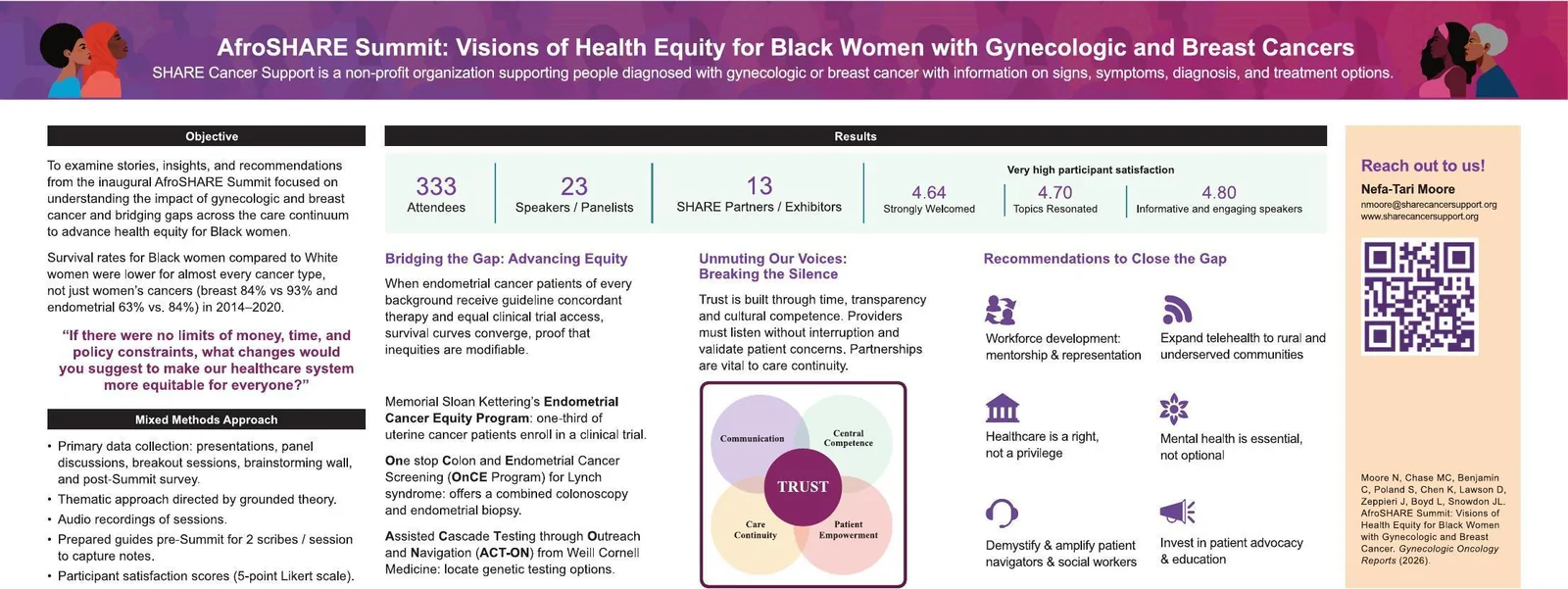

## 1 Introduction

Women's cancer incidence rates are rising in the United States with one million new diagnoses/year: breast (32%) is most common and uterine corpus (7%) fourth (*American Cancer Society. Cancer Facts, and Figures 2026. Atlanta, GA,* 2026). Survival rates for Black women compared to White women were lower for almost every cancer type in 2016–2022 (National Cancer Institute Surveillance, Epidemiology and End Results Program, 2026). Five-year relative breast cancer survival rates are 84% (Black) and 93% (White) (*American Cancer Society. Cancer Facts, and Figures 2026. Atlanta, GA,* 2026; National Cancer Institute Surveillance, Epidemiology and End Results Program, 2026). Endometrial cancer overtook all gynecologic malignancies as the most lethal with the burden falling hardest on Black women because of: higher prevalence of biologically aggressive, non-endometrioid tumors; late diagnosis; patients may not receive needed biopsies due to disadvantages in social determinants of health; and less access to high-quality screening and treatment (*American Cancer Society. Cancer Facts, and Figures 2026. Atlanta, GA,* 2026; National Cancer Institute Surveillance, Epidemiology and End Results Program, 2026; *U. S. Department of Health and Human Services, Centers for Disease Control and Prevention (CDC). Uterine Cancer Facts. CDC Publication* #99–9125, 2026; C. Brown. Bridging the Gap, 2025).

This trend is a widening equity crisis fueled by intertwining patient and healthcare system factors (Clarke et al., 2022; Ward et al., 2008). Towner et al. present a theoretical framework explaining disparities between White and Black women's gynecologic outcomes (Towner et al., 2022). Rodriquez et al. report disparities for women with endometrial cancer in timeliness of care and equitable access even after adjusting for sociodemographic and clinical factors (Rodriquez et al., 2025).

This study explores the lived experiences, themes, and community-driven recommendations from the inaugural AfroSHARE Summit focused on gynecologic and breast cancers and their disproportionate impact on Black women held on June 24, 2025. The Summit aimed to bridge systemic gaps across the care continuum and catalyze health equity. Guided by the central research question, “If there were no limits of money, time, or policy constraints, what changes would you propose to create a more equitable healthcare system for all?”, this study synthesizes qualitative insights from participants to inform future policy and community-based strategies for transformative change.

## 2 Methods

### 2.1 Study organization

SHARE Cancer Support (SHARE), https://www.sharecancersupport.org, is a nonprofit organization supporting people diagnosed with gynecologic or breast cancer with information on signs, symptoms, diagnosis, and treatment options. Annually 350,000 people worldwide have benefited from SHARE's free-of-charge services: expert-led live webinars, national helplines, online communities, educational tools, clinical trial assistance, outreach/education in socioeconomically disadvantaged communities, and patient navigation services.

### 2.2 Ethics approval

All speakers provided written consent to audio-recording Summit sessions.

### 2.3 Study design and end points

A mixed methods approach was used to understand the needs of Black women with gynecologic/breast cancer and to uncover practical recommendations for delivering services effectively to improve well-being and health equity. Primary study end points were qualitative and quantitative measures. Qualitative data were obtained through presentations, panels, breakout sessions, the brainstorming idea wall, and a post-Summit survey. Qualitative measures included participant perspectives on barriers faced across the cancer care continuum organized into advocacy, community, education, and justice categories. A thematic approach directed by grounded theory was employed to collect participants contributed ideas regarding the overarching research question, which were written on sticky notes and applied to a brainstorming wall, analyze the ideas for patterns, and categorize them into themes (Chapman et al., 2015). Two research team members systematically performed the coding of ideas and any disagreements were resolved through discussion until consensus was reached. Quantitative measures, such as participant satisfaction scores (5-point Likert scale), were analyzed from post-Summit survey responses.

The Summit was recorded using Zoom Workplace software (*Zoom Workplace. V6.4.*12, 2025). Using guides prepared pre-Summit, two scribes per breakout session captured notes. Descriptive statistics for each outcome of interest were calculated using Microsoft Excel.

## 3 Results

### 3.1 Summit overview

The AfroSHARE Summit convened 333 (122 in-person, 211 livestream) attendees, twenty-three speakers/panelists, and thirteen partners/exhibitors. Nefa-Tari Moore, SHARE's Director of Black Women's Outreach and 2025 Dicey Scroggins Distinguished Advocacy Awardee, kicked off the event. Serrina Goodman, AfroSHARE Ambassador Coordinator offered a spiritual moment. Heidi Henderson, SHARE's Executive Director and CEO presented “From Symptoms to Stories: Understanding Gynecologic and Breast Cancers” and played an informative video.

### 3.2 Bridging the gap: advancing equity

Dr. Carol Brown, a gynecologic oncologist, Senior Vice President and Chief Health Equity Officer at Memorial Sloan Kettering Cancer Center (MSK) and Vice Chair for Health Equity in the Department of Surgery, delivered a powerful keynote highlighting increasing endometrial cancer deaths (1.8%/year) and the largest racial disparities in outcomes with 5-year relative survival rates for Black women (63%) compared to White women (84%) (*American Cancer Society. Cancer Facts, and Figures 2026. Atlanta, GA,* 2026; National Cancer Institute Surveillance, Epidemiology and End Results Program, 2026; C. Brown. Bridging the Gap, 2025; Clarke et al., 2022). Quoting Dr. Martin Luther King Jr. “injustice in health care is the most shocking,” Brown rallied “we must pursue genuine health equity.”

Tumor biology and systemic barriers (e.g., limited access to high-volume gynecologic-oncology centers, insurance and income gaps, historic mistrust, low clinical-trial participation) matter. Even when disease stage is comparable, outcomes diverge once care is delivered in low-volume settings without multidisciplinary expertise. MSK's data reveal when patients of every background receive guideline-concordant therapy and equal trial access, survival curves converge, proof that inequities are modifiable (C. Brown. Bridging the Gap, 2025; Memorial Sloan Kettering Cancer Center, 2021; Manning-Geist et al., 2022; Weigelt et al., 2023). MSK's Endometrial Cancer Equity Program attacks the gap with culturally tailored education and streamlined referrals to promptly biopsy abnormal bleeding, research in comprehensive genomic profiling to unravel why biologically aggressive, non-endometrioid cancers cluster in Black women and to identify actionable targets, and training through bias-mitigation workshops and pipeline initiatives that diversify the workforce. A tangible result is one-third of MSK's uterine-cancer patients enroll in a clinical trial.

Brown concluded with three messages. Equality doesn't mean equity. “In cancer and other chronic diseases, we have to recognize some people inherently have worse outcomes and find out why and give them what they need to achieve equity.” Stay current on screenings, advocate for yourself, and explore clinical-trial options if diagnosed. Abnormal uterine bleeding warrants an endometrial biopsy, no exceptions.

### 3.3 Unmuting our voices: breaking the silence

Social worker Cynthia Chalker moderated two panelists Nefa-Tari Moore and Megan-Claire Chase, SHARE's Breast Cancer Program Director and Podcast Host, who told moving, inspirational stories about disparities and stigmas associated with their identities and cancer treatment experiences.

#### 3.3.1 Lynch syndrome impacted care

Moore, a young Black Muslim three-time uterine and ovarian cancer survivor/patient advocate, discovered she had Lynch syndrome, an inherited genetic mutation associated with increased genetic risk of colon, endometrial, and other cancers. Her testimony highlighted patient education, mental health services, self-advocacy, and the need for collaborative action to work towards equitable outcomes.

**Pre-Diagnosis:** Moore experienced abnormal bleeding, lower back pain, and energy loss. Doctors dismissed her symptoms and incorrectly concluded Moore had a sexually transmitted disease or was pregnant. Moore's test results appeared negative so doctors diagnosed, without performing a physical examination, the bleeding was normal due to a menstrual cycle change and stress, and prescribed Ibuprofen, which can cause potential bleeding. Being dismissed, not being examined, and testing were rooted in discrimination and racism. Determined to get better answers, a transvaginal ultrasound showed the source of her bleeding.

**Post-Diagnosis:** Moore was diagnosed with ovarian cancer and Lynch syndrome. She felt relieved and empowered to learn why she'd been diagnosed three times with cancer throughout her thirties. Moore's family was motivated to get screened, some opting for genetic testing, which brought the family closer.

Moore was afraid of dying and experienced depression, sleepless nights, and crying bouts. Moore's oncologist rightfully focused on her cancer diagnosis. Inquiries should have been made about her emotional well-being. One morning during religious preparations, Moore felt newfound determination to live and self-advocate. She tried finding support groups, but found none specific to endometrial cancer. Moore turned to her faith, family, and friends for support.

After a year of treatment Moore was cancer-free. Less than one year later, she had a uterine cancer recurrence and partial hysterectomy, uterus removed only. During the post-operative appointment, Moore learned her oncologist/surgeon left the practice so she found a new specialist. Through a routine checkup, Moore was diagnosed with ovarian cancer, had an oophorectomy, and re-entered treatment. This time her care team acted with compassion and respected her dignity by addressing her physical and emotional health and providing supportive resources. Moore's oncologist was a Black woman: “I think representation is key for building trust and health equity.”

#### 3.3.2 Being diagnosed with breast cancer under 40

Chase, a Black breast cancer survivor/patient advocate, was diagnosed under age 40. Her story highlighted systemic inequities and the urgency for culturally competent, patient-centered care and representation in educational materials.

**Pre-Diagnosis:** Chase's cancer journey began with subtle, persistent changes in her body including unexplained weight gain, changes in her hair, and a normal mammogram. She was repeatedly dismissed by medical professionals who advised “just eat better,” “you're fine,” and “come back when you're 40.” Two years later Chase discovered a hard lump in her left breast and underwent a biopsy.

**Post-Diagnosis:** Chase was diagnosed with invasive lobular breast cancer, which can be challenging to detect on mammography, especially in dense breasts, which Chase had. Her mother was simultaneously receiving blood cancer treatment. They served as each other's caregivers. This dual role created emotional strain. More supportive services for families navigating simultaneous cancer diagnoses are needed, for example, integrating clinical management, psychosocial support, and patient-centered guidance for optimizing care delivery for the family. Despite the gravity of Chase's 16 chemotherapy sessions, the fertility preservation option was presented without meaningful discussion or follow-up. Chase dealt with a dismissive oncologist, significant insurance challenges, and financial struggles. Chase thanked her oncologist for getting her to active treatment completion; however, “you're not listening to me anymore, so I divorce you.”

Chase endured financial toxicity. The social worker assigned to her oncologist was pivotal in guiding Chase to apply for grants for cancer treatment and essential living expenses. “This is cancer, not the flu. This is the time to swallow your pride and to ask for help.” Chase relied on crowdfunding to manage unexpected costs.

Chase experienced the inadequacy of pre-cancer coping tools upon entering survivorship and lack of mental health providers who understood racial trauma. She recounted a breakthrough after being initially skeptical about working with a young, white male therapist who ultimately gave her new coping strategies: “I had to audition therapists – it's hard to find someone who really gets what this experience is like for a Black woman.”

Lack of long-term financial support in the survivorship stage is challenging. Critical physical or occupational therapy services and newer medications and treatments are often only partially, or not at all, covered by insurance, placing an ongoing financial burden on patients navigating life after active treatment.

#### 3.3.3 Patient insights and recommendations

Word clouds in Fig. 1 show Moore's (left) and Chase's (right) patient experiences and resilience (Zygomatic, 2025). They offer valuable guidance in Fig. 2 to address not just the disease, but the whole person.

**Fig. 1:**
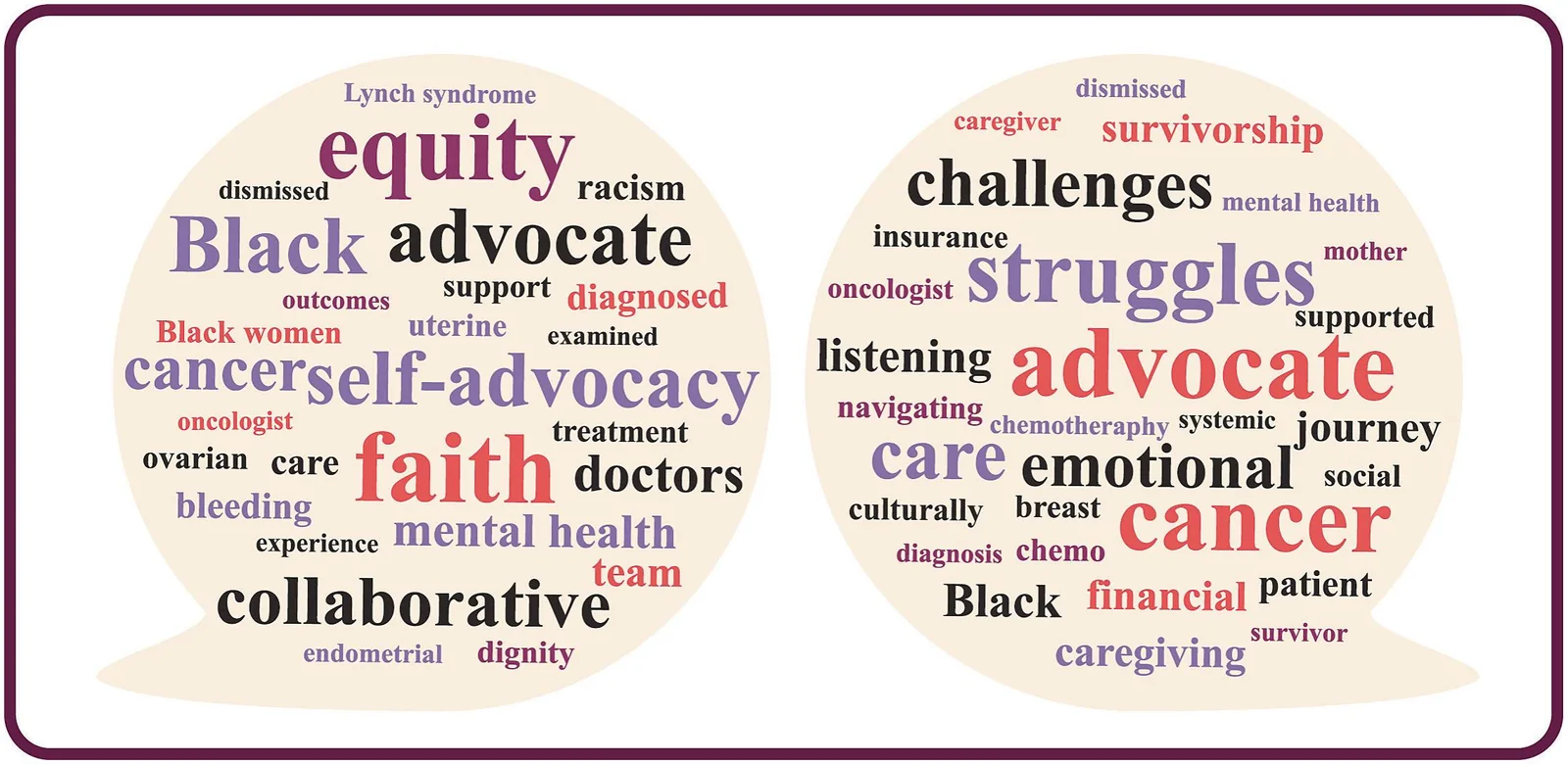
Black Cancer Patients' Experiences.

**Fig. 2:**
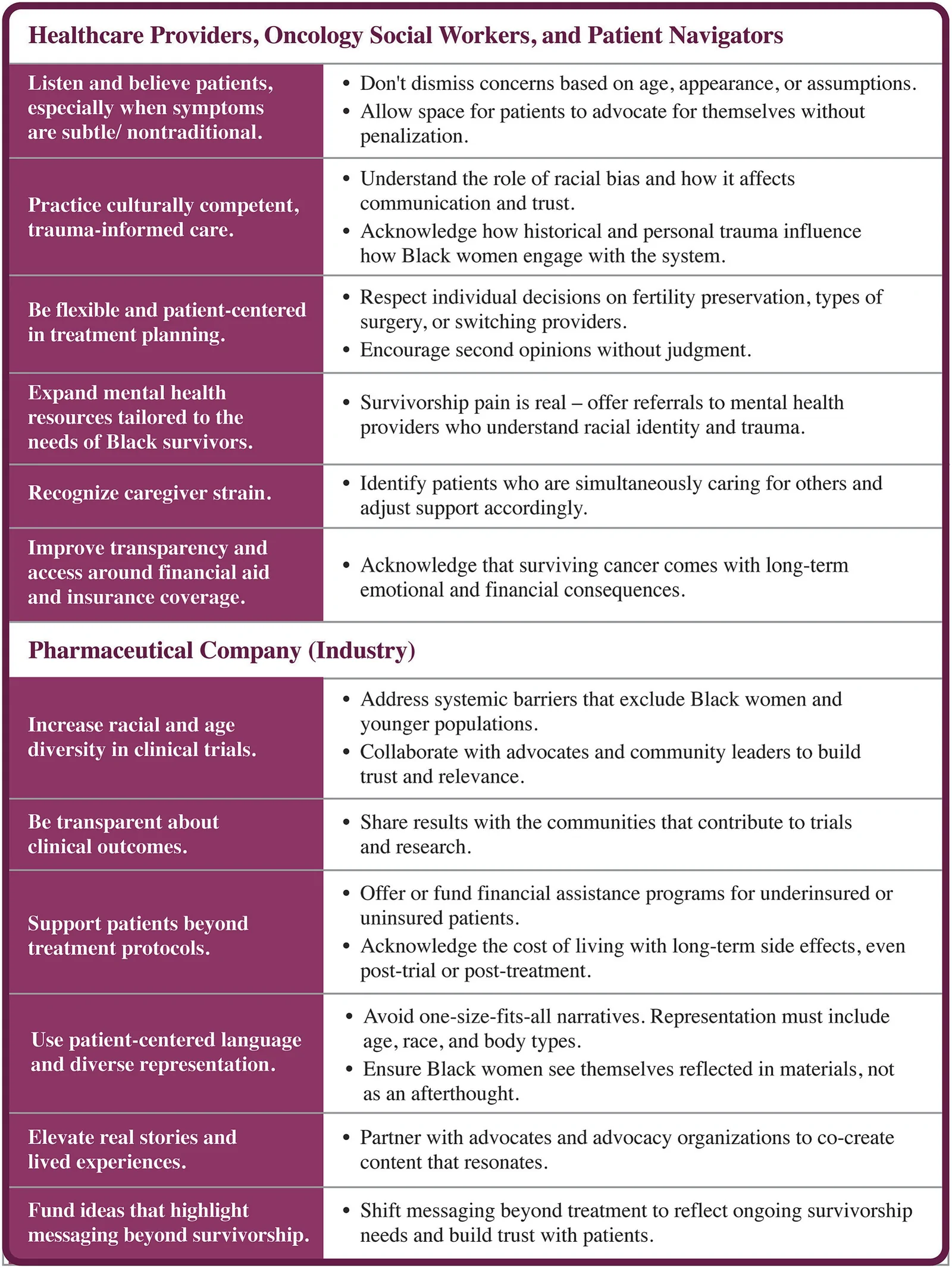
Black Cancer Patient Insights and Recommendations for Providers and Pharmaceuticals.

### 3.4 Building trust

Moderator Godsfavour Guillet, Mount Sinai's Director of Hospital and Nursing Administration, explored Black patients' painful realities navigating healthcare. Four panelists, a nurse and metastatic breast cancer patient/advocate, Kimberly Vann, an endometrial cancer survivor/advocate, nurse practitioner Latasha Anderson-Dunkley, and Dr. Leslie Boyd, Director of Gynecologic Oncology at NYU Langone, identified five key themes. “Erosion of trust in the healthcare system” is born from repeated medical dismissals, systemic failures, and associated trauma. “Strategies in building trust” are fragile and rooted in every interaction. “The power of second opinions: a critical act of self-advocacy” is a patient right. “The power of listening and representation” improves outcomes and patient experiences, fosters trust in care teams, and encourages sharing results transparently. “Mental health must be normalized,” said Guillet, “When you are listened to, you open up like a flower.”

Fig. 3 illustrates the overlapping roles of communication, cultural competence, care continuity, and patient empowerment as pillars supporting patient trust. Trust is built through time, transparency and cultural humility. Providers must listen without interruption and validate patient concerns. Diverse representation among providers improves patient comfort and outcomes. Normalizing mental health and second opinions empowers patients. Community partnerships are vital to care continuity.

**Fig. 3:**
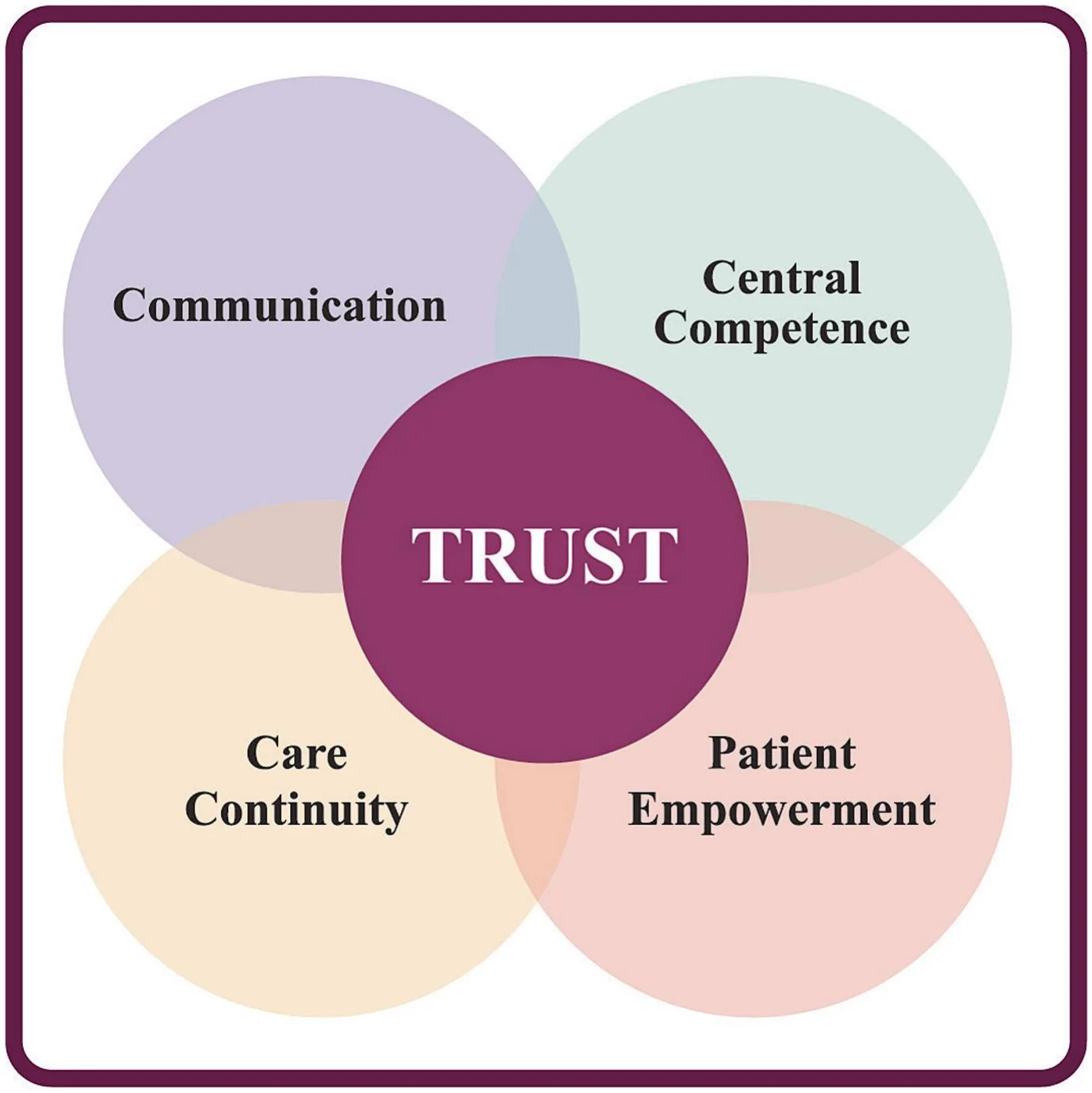
Key Drivers of Trust in the Black Patient Experience.

### 3.5 Protect your peace

Moderator Kettura Campbell, AfroSHARE Programs Associate, conversed with Jennifer LaPietra, Oncology Social Worker and Patient Assistance Program Manager at CancerCare, about creating boundaries, balance, and breaks to address the weight carried by cancer patients and their loved ones. LaPietra provided tools and ideas for emotional healing during stressful times.

#### 3.5.1 Burnout and fatigue

Exhaustion from chronic stress and compassion fatigue risks impacting overall health and may lead to burnout for cancer patients' loved ones. Common burnout warning signs may be overlooked, such as irritability, frequent emotional outbursts, trouble sleeping, social isolation and avoidance, and weight changes. Cancer patients may also experience some of these symptoms as well as fatigue caused by body pain or gastrointestinal issues from cancer or treatment side effects.

LaPietra introduced the Fatigue Wheel and asked attendees to rank their exhaustion in the past 24 h and “How do you navigate the fatigue when you are exhausted?” (Cancer Fatigues Services Inc., 2025). She recommended honestly talking with medical professionals about side effects, exploring treatment plan adjustments if needed, asking about integrative care, palliative care, nutritional support or therapy to manage symptoms or side effects, accepting emotional support for individual counseling or support groups, keeping communications open, and learning about stress reduction tools.

A caregiver expressed take care of yourself first when serving as a patient's first line of support and don't feel guilty. If family is not the best support system, then look to friends and community services.

#### 3.5.2 Boundaries

Quoting Tawwab (Tawwab, 2021), LaPietra defined boundaries as “expectations and needs that help you feel safe and comfortable in relationships.” Boundaries inform others “HOW to show up for you” to avoid becoming overwhelmed, irritated, or taken for granted.

LaPietra described a wheel with seven boundary types: mental, emotional, material, internal, conversational, physical, and time. When respectful boundaries are not set, meaningful, healthy connections can be impacted. LaPietra explained the importance of saying “no” and explaining why. An attendee suggested saying “I am sharing this out of love.” Another added “when we think they should know, they do not.”

#### 3.5.3 Values and self-care

LaPietra recounted “Values are principles you believe are most important. They help determine priorities, behavior, and what gives your life meaning.” A cancer survivor shared how their diagnosis makes it difficult to prioritize things they value: career, sense of beauty, and relationships. A pivot happened when focusing on their purpose again and reevaluating values.

Spirituality can be helpful during the uncertainty of diagnosis. Participants emphasized prioritizing health and learning to relax expectations and ambitions as part of “reimagining life.”

Self-care varies from taking walks, to spending time with pets, to mindfulness and stress reduction techniques, to creating space for emotional release. One survivor felt guilty for not following their planned self-care routine, nor showing gratitude, later realizing that rest by “sitting on the couch and just being” is also self-care. LaPietra advised “Check in with yourself regularly, our needs change, our bodies are consistently processing, and we might need replenishment in different areas.”

#### 3.5.4 Hope and purpose

Hope is the foundation on which we build our wellness. It is our most vital emotion; it is what “gets us through our darkest time.” Purpose is often found in our passions, those things that light us up, energize us, and make us feel deeply alive. LaPietra shared tips for protecting your peace: helping others, prioritizing self-care, exploring spirituality, engaging in lifelong learning, advocating for change, and embracing adventure. LaPietra concluded with a mindfulness meditation and three takeaways: it is okay to share what you need, boundaries are for you, not for anyone else, and setting boundaries can require mindful conversation.

### 3.6 Advocacy

Moderator Natasha Coleman, Vice President of Community Impact at the American Cancer Society (ACS), Northeast Region, conversed with Megan-Claire Chase, a breast cancer survivor who previously described her story in Section 3.3 and three additional panelists including Aisha McClellan, SHARE's Cervical Cancer Program Coordinator and cervical cancer survivor, Annie Ellis, ovarian cancer survivor and research advocate, and Gwendolyn Harrison, AfroSHARE Ambassador, National Breast Cancer Coalition (NBCC) New York City Leader and breast cancer survivor. They found their voice through storytelling, research advocacy, legislative engagement, and peer-to-peer support. Advocacy is not one-size-fits-all; it must align with your personal values, bandwidth, and passion.

McClellan talked about cervical cancer stigma and how peer support and social media became her entry points into advocacy: “Your story matters.” Harrison emphasized the impact of legislative advocacy and training: “Research advocacy chose me.” Ellis spoke about research advocacy and elevating patient's perspectives in clinical conversations: “Sharing your experience, telling our truths.” Chase shared how storytelling and creative expression helped her move through pain into purpose.

Advocacy can be segmented into four categories. Research advocacy ensures the lived experience of patients shapes clinical trials and scientific inquiry. Storytelling and writing convey personal narratives to raise awareness, combat stigma, and build community. Social media advocacy connects, educates, and mobilizes others online. Organizational advocacy supports mission-driven nonprofits through program work, outreach, and peer mentoring.

Panelists named racism, representation gaps, and systemic healthcare inequities as major advocacy drivers. They urged advocates to speak up about racism in clinical settings, within support spaces, and to validate mental health struggles. Advocacy has an emotional toll. “You have to pour yourself before you pour into others.” “Connection gives you joy, but triggers can happen anytime.”

Panelists suggested a five-step process for advocating: identify your passion, assess your capacity, find a mentor, set boundaries, and take care of your mental health. Participants were encouraged to consider their own skills and capacity and to begin exploring where their voice fits.

### 3.7 DNA and dignity

Moderator Kitty Silverman, SHARE's Uterine and Endometrial Cancer Program Director, spoke with Dr. Jennel Zeppieri, a medical oncologist specializing in cancer genetics, prevention, and high-risk breast cancer survelliance at Weill Cornell Medicine, about the role of genetic testing and data-driven approaches in assessing and managing cancer risk. Zeppieri emphasized genetic testing and counseling empower patients to make informed decisions about cancer prevention strategies. For patients with existing cancer, identifying genetic mutations can have important therapeutic implications.

Up to 5% of the population carries a hereditary mutation associated with increased cancer susceptibility, and roughly 25% are candidates for genetic testing. Testing is available to all adults and may be considered in children when both parents carry mutations that increase the risk of rare conditions, such as Fanconi's Anemia. As genetic testing panels evolve, individuals who previously tested negative should consider repeat testing using updated, clinically appropriate panels.

Up to 10% of breast cancers are linked to germline mutations, including BRCA1, BRCA2, PALB2, CHEK2, and ATM. Black women at high risk of breast cancer are less likely to be referred to a specialist for risk management when compared to white women and among the referrals, only 15% of black women were seen by the specialist versus 70% of white women. Because overall breast cancer risk is influenced by factors beyond genetic mutation, patients are encouraged to discuss breast density, magnetic resonance imaging, and validated risk-assessment tools such as the Tyrer-Cuzick and Gail models with their healthcare providers to support personalized evaluation. Individuals without genetic mutations but at high risk of breast cancer may also be eligible for medications that can significantly reduce risk.

Germline mutations, including BRCA1, BRCA2, and Lynch Syndrome-associated genes, account for 20–25% of ovarian cancers and 5% of uterine cancers. Most (75%–90%) individuals with endometrial cancer present with abnormal uterine bleeding. Routine uterine cancer screening is not recommended due to insufficient evidence for mortality reduction. Individuals with Lynch syndrome may consider endometrial biopsy every 1–2 years starting at ages 30–35, prophylactic total hysterectomy, and transvaginal ultrasound for postmenopausal women.

#### 3.7.1 Health equity and access

Race should not be used to determine who should be referred to genetic testing. Outreach within Black communities through virtual appointments and digital cancer risk assessments that accommodate conflicting schedules and locations is expanding access and helping to address disparities.

Empowerment is cultivated through open, respectful conversations and effective health education delivery provided by professionals who reflect the populations and communities they serve. One participant noted “You might not get people to listen if you don't have someone who looks like them,” highlighting the critical need for more Black health professionals. Advocates and professionals should consider working with local community leaders and representatives to endorse and communicate necessary information on confidentiality and safety for wider trusted reach.

#### 3.7.2 Programs and support

Up to 95% of Americans harboring cancer-predisposing germline pathogenic variants have not been identified despite recommendations for screening at the primary care level (Bull et al., 2024). **He**alth **R**isk **I**nformation **T**echnology – **A**ssisted **G**enetic **E**valuation (**HeRITAGE**), a randomized controlled trial of a digital genetic cancer risk stratification tool in an urban and diverse underserved gynecology clinic, resulted in increased identification and counseling of high-risk patients recommended for genetic testing (Webster et al., 2025).

Patients are encouraged to explore their eligibility for:

1. **On**e stop **C**olon and **E**ndometrial Cancer Screening (**OnCE** Program) for Lynch syndrome offers a combined colonoscopy and endometrial biopsy, which provides many advantages over getting screened separately.
2. **A**ssisted **C**ascade **T**esting through **O**utreach and **N**avigation (**ACT-ON**) offers family members of people with hereditary cancer mutations support and assistance to locate genetic testing options accessible to their location.

### 3.8 Truth, trust, and trials

Moderator Aisha McClellan, Cervical Cacer Program Coordinator at SHARE, unpacked the roots of medical mistrust in Black communities with panelists Uzo Osameka, Programs and Community Lead, TOUCH, The Breast Cancer Alliance, Dr. Onyinye Balogun, Assistant Professor of Radiation Oncology, Weill Cornell Medical College, and Karen Peterson, Founder and Chief Patient Advocate, Karen's Club. Balogun urged a shift in perspective from community mistrust to “What is the medical establishment doing to prove themselves trustworthy?” Balogun provided an overview of clinical trial phases (I-III) and how each step is vital to testing treatment safety and efficacy. The institution's tumor board presents every patient's case for clinical trial opportunities. “I advocate for infrastructure. Without it, we can't run trials that include Black and Hispanic people.”

Peterson navigated the unknown when diagnosed with metastatic triple-negative breast cancer, which disproportionately affects Black women at rates 2–3 times higher than White women and results in a one-third higher risk of mortality (Ensenyat-Mendez et al., 2023). She explained “I went through a lot to survive. Clinical trials were not presented to me. It was like the Wild West. I was talking with doctors at conferences and sneaking into research labs … These are the things I had to do to stay alive.” Through biomarker testing, Peterson discovered she was eligible for an investigational immunotherapy trial. After eight weeks on the trial, her lesions shrank by 72%. She has been in remission for seven years: “That biomarker saved my life.”

Misconceptions can hinder clinical trial participation, e.g., “I don't want to be a guinea pig; they're testing something unfounded on me” said Osameka. Balogun countered “You often are getting a higher level of care.” Peterson warned “The window of opportunity is short. Co-morbidities or prior treatments can make you ineligible.” These beliefs are compounded by socioeconomic disparities, unequal access to cutting-edge treatments, and even biological differences; all contribute to survival gaps (Halpern et al., 2008; Danforth Jr., 2013; Obeng-Gyasi et al., 2021; Sturtz et al., 2014).

Peterson is a community scientist with the Tisch Cancer Institute where she helped 78% of participants she engaged enroll in trials – demonstrating the power of peer-led, culturally competent outreach (National Academies of Sciences, Engineering, and Medicine, et al., 2022; Swanson and Ward, 1995). “I translate what patients say into something researchers can use. We go to where the people are…where they live, work, play, and slay.”

Osameka closed with “Think of clinical trials as an investment in your health or a ‘medical 401(k)’ … a trust fund for your babies.” Peterson stated “Clinical trials are an opportunity. You never know where that conversation will lead.” Balogun concluded with “Change starts with me. If we want to see more Black women in trials, consider being *the* Black patient in a trial.” Key takeaways were to shift the narrative, normalize clinical trials, meet communities where they are, create institutional infrastructure, use peer educators and community scientists, and empower patients through education.

## 4 Post-summit survey

The post-Summit survey response rate was 13.2% (44/333), which is low. However, the overwhelming positive responses are still important to note. Three survey questions assessed perceived usefulness and were scored on a 5-point Likert scale where 1 = strongly disagree and 5 = strongly agree. Participants indicated very high satisfaction with perceived feeling of being strongly welcomed at the Summit (4.64), Summit topics resonating with them (4.70), and Summit speakers being informative and engaging (4.80). Fig. 4 illustrates the key takeaways survey participants submitted in free-form text input organized according to advocacy, community, education, and justice categories.

**Fig. 4:**
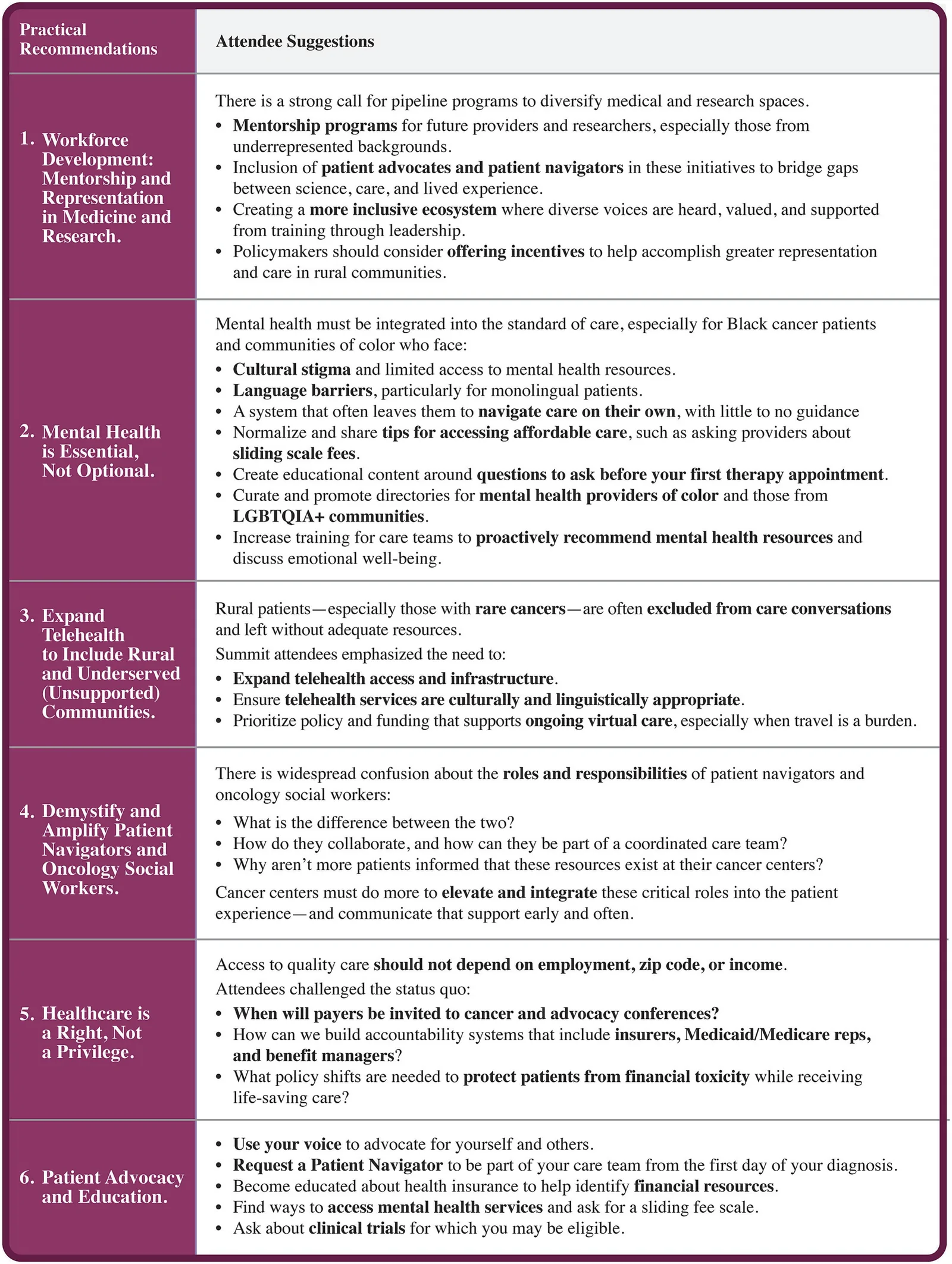
Post-Summit Survey: Key Takeaways.

## 5 Discussion

The Summit's core theme “**Believe, Listen, and Support Black Patients”** and six practical recommendations and associated attendee suggestions from the brainstorming idea wall are summarized in Fig. 5. Healthcare systems, providers, and researchers must **believe** Black patients and truly **listen** when they share their experiences, which often are met with racism or racial bias, dismissiveness around pain or concerns, or microaggressions from medical staff. The burden should be shifted away from patients to prove their experiences. Trust must be built and upheld by the system itself. Fig. 6 gives a set of recommendations developed for key stakeholders to help close disparities' gaps. For example, more advocacy is needed to expand Medicaid and other services to provide greater Social Security disability coverage for people with cancer, as in the Metastatic Breast Cancer Access to Care Act, (2026).

**Fig. 5:**
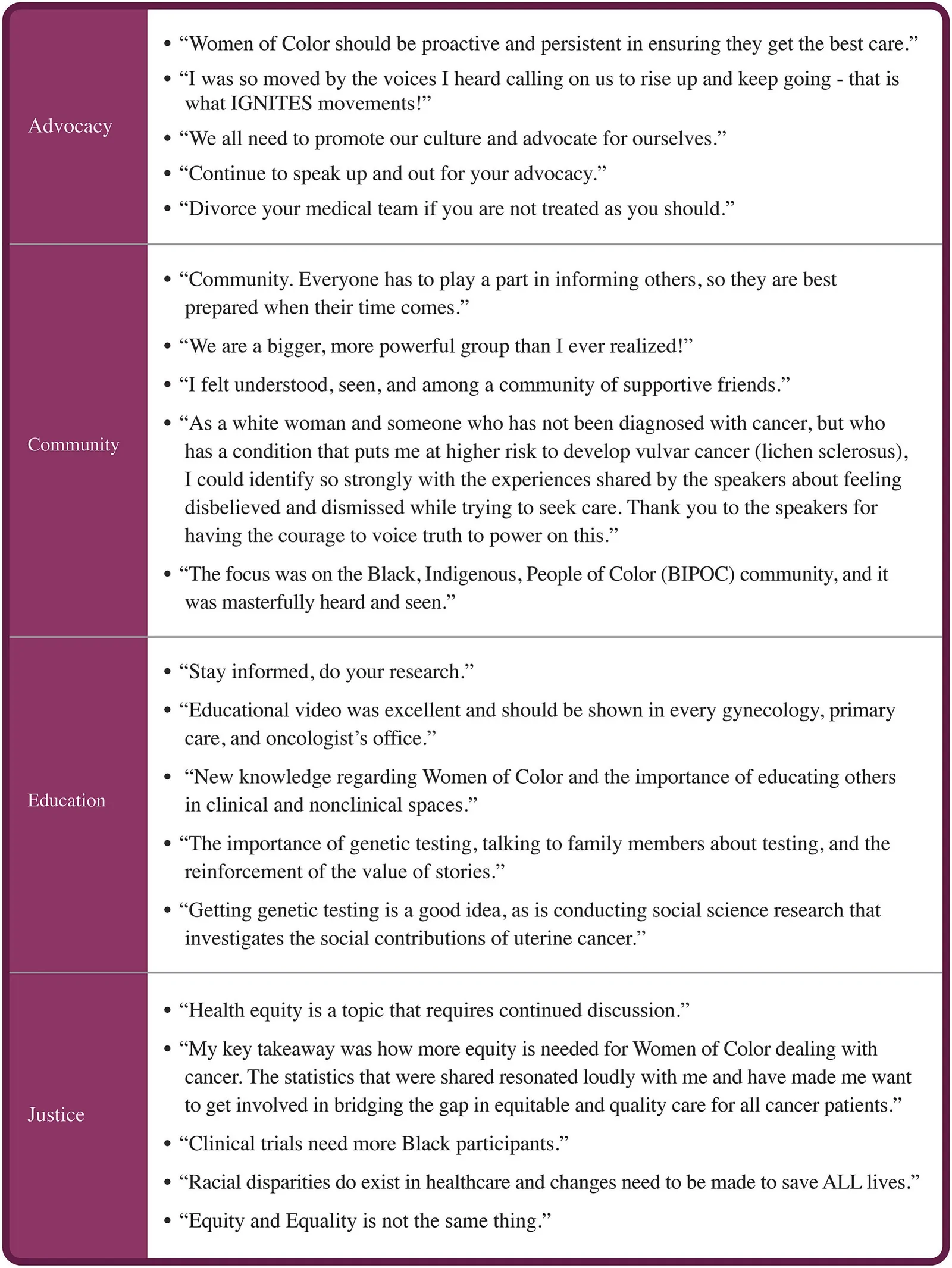
Summary of Practical Recommendations and Attendee Suggestions.

**Fig. 6:**
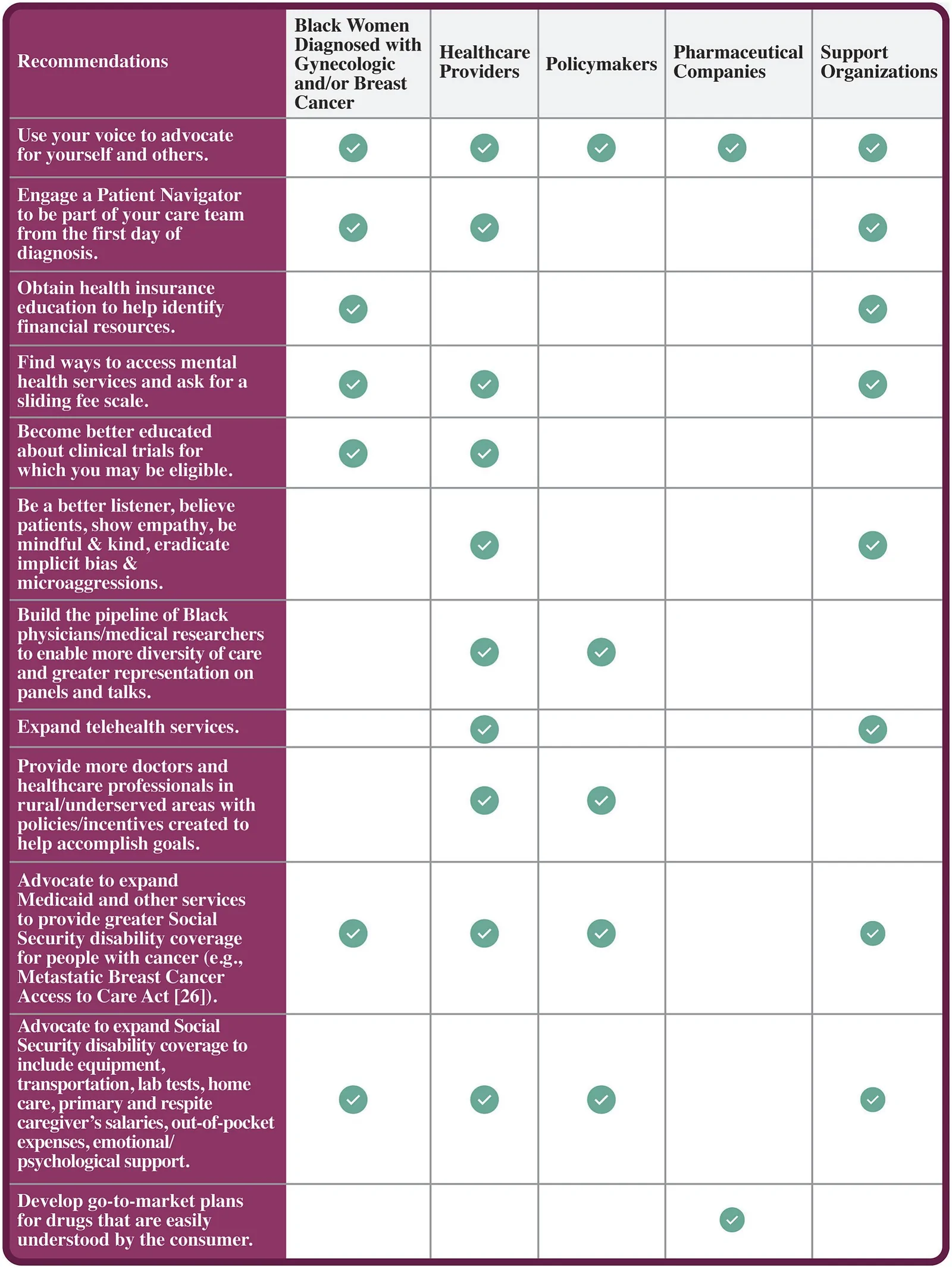
Recommendations for Closing the Disparities' Gaps for Stakeholders and Black Women with Gynecologic and/or Breast Cancers.

## 6 Conclusions

Bringing together voices from across the gynecologic and breast cancer care continuum enabled better understanding of the health care experiences, support, and information needs of Black women and their families, and generated practical recommendations for delivering services effectively and efficiently. Together we can build a future of empowerment, community, and impact.

## CRediT authorship contribution statement

**Nefa-Tari Moore:** Writing – review & editing, Writing – original draft, Formal analysis, Conceptualization. **Megan-Claire Chase:** Writing – review & editing, Writing – original draft, Formal analysis, Data curation, Conceptualization. **Christine Benjamin:** Writing – review & editing, Writing – original draft, Formal analysis, Conceptualization. **Stephanie Poland:** Writing – review & editing, Writing – original draft, Formal analysis, Conceptualization. **Kaiyun Chen:** Writing – review & editing, Writing – original draft, Formal analysis, Data curation. **Denise Lawson:** Writing – review & editing. **Jennel Zeppieri:** Writing – review & editing. **Leslie R. Boyd:** Writing – review & editing. **Jane L. Snowdon:** Writing – review & editing, Writing – original draft, Supervision, Formal analysis, Data curation.

## Ethics declaration

Written informed consent to take part in the study and to publish the article has been obtained from all participants or their legal representatives. The privacy rights of participants have been observed.

This study was performed in compliance with relevant laws, regulatory frameworks and guidelines where the research took place.

This study was approved by SHARE Cancer Support.

(Approval No. Approved by the SHARE Cancer Support Board of Directors)

## Funding

This research did not receive any specific grant from funding agencies in the public, commercial, or not-for-profit sectors.

## Declaration of competing interest

The authors declare the following financial interests/personal relationships which may be considered as potential competing interests: Leslie Boyd, MD reports a relationship with Astra Zeneca that includes: consulting or advisory. Leslie Boyd, MD reports a relationship with ASCO Advantage Lecture Series that includes: speaking and lecture fees. The other authors declare that they have no known competing financial interests or personal relationships that could have appeared to influence the work reported in this paper.
